# A scoping review of the nature of physiotherapists’ role to avoid fall in people with Parkinsonism

**DOI:** 10.1007/s10072-020-05015-y

**Published:** 2021-01-14

**Authors:** Salem F. Alatawi

**Affiliations:** Associate professor of neurorehabilitation Department of Physical Therapy Faculty of Applied Medical Sciences, University of Tabuk, Tabuk City, Saudi Arabia

**Keywords:** Parkinson’s disease, Physiotherapy, Rehabilitation, Exercise, Falls, Risk factors

## Abstract

**Background:**

Parkinson’s disease (PD) is considered a neurological disease with a high prevalence rate among population. One of its main problems is recurrent fall which has numerous contributing factors such as history of fall, fear of falling, gait deficits, impaired balance, poor functional mobility, and muscle weakness.

**Objective:**

To review and explore the focus/nature of interventions which target the role of physiotherapy preventing fall in patients with PD.

**Method:**

A scoping review was led dependent on Arksey and O’Malley as discussed by Wood et al. (2002). This paper based on this structure to perceive intervention studies have been embraced in physiotherapy to prevent fall after Parkinson’s disease. The search included various databases. The referencing arrangements of every pertinent paper were additionally filtered for more studies.

**Findings:**

A total of 173 articles were included, 39 of which met the eligibility criteria. Fifteen studies reported on the direct impact of physiotherapy on fall, while the rest examined the impacts of physiotherapy on factors that are associated with fall. Different outcomes, interventions types, and duration were used in these studies. Findings showed a favorable result of physiotherapy on fall and near fall incidence, balance, gait, functional mobility, muscle strength, and fear of falling.

**Conclusion:**

Physiotherapy has the possibility to decrease fall incidence and fall risk in people with PD. However, the heterogeneity in the patients’ selection, intervention studies, outcome measures chosen, time since the onset of disease, variation in intensity, and duration of treatment between included studies make the comparisons difficult. Consequently, more studies are needed on best intervention.

## Background

Parkinson’s disease (PD) is a common, exhausting, and progressive neurodegenerative disease. The elderly suffers from falls as part of the aging process, and PD patients have this as one of its complications [1].

Studies regarding the issue of falling found that those who are suffering from PD are more prone to falls compared either with healthy people of the same age or with the elderly who have other neurological conditions [2, 3]. A study by Bloem et al. [2] conducted on fall prospectively in people with PD and in elderly people with good health found that PD results in a 9-fold increase in the likelihood of experiencing recurrent falls compared with healthy people of the same age. In Stolze et al.’s study (2004) [3], in comparison to patients with neurological disease “like stroke,” the Parkinson’s patients have two times risk of fall.

The incidence rate of falls through the Parkinson’s patients ranged between 38 and 87% [4, 5]. In a study done by Hely et al. [6], measuring the risk of fall during 20 years of the Parkinson disease, 87% of them will experience falling at least one time. It was additionally discovered that falls in PD are intermittent. Pickering et al. [7] found that roughly 15% of individuals with this disease involvement have at least one fall each week, while different investigations announced that half of those with PD fell twice or more throughout 1 year. In this manner, a high number will experience the complications of falling.

Recognizing the fall risk factors is of vital importance in fall prevention. As of late, a lot of research has been directed into the reasons for falls, and a substantial number of variables have been found.

A systematic review and meta-analysis tried to summarize the evidence regarding fall risk factors in people with PD [7]. However, the authors of this meta-analysis reviewed only six fall studies and found that the most solid factor which could anticipate future falls is a history of fall. However, in spite of the fact that this factor supports the belief that fall in PD is intermittent and individuals with a previous history of fall are at extraordinary hazard and should be dealt with, this factor is not treatable in that knowing the history of fall leads to more falling can help people with PD to take preventative measures and does not show the reason for the first event of fall. Therefore, concentrating on other treatable elements related with fall is of incredible significance in the avoidance of the main fall, and should in this way be viewed as while assessing the impacts of any intervention or when designing an intervention to prevent fall. Studies exploring the reasons for fall have found different factors strongly related with falls and have recommended rehabilitation programs to focus on these components to prevent fall in individuals with PD. The most important factors include reduced balance, impaired gait, muscle weakness, poor functional mobility, or performance on functional tasks, and, in addition to these physical limitations, psychological factors are also found to be risk factors [4].

Physiotherapy is one of the most important approaches of treatment for people with PD. It has continually been observed to be a viable method to alter factors, for example, balance and gait, as to diminish the quantity of falls among the elderly [5].

Various reviews [5, 8–10] have been done to condense the proof and to decide if physiotherapy interventions are useful for individuals with PD. However, most of these reviews did not concentrate on falls, but instead, they researched the general potential advantages of physiotherapy intervention on individuals with PD to the extent that only one systematic review focused on fall. In this specific review, Allen et al. [10] searched the literature up to 2009 and directed a meta-analysis to decide the impacts of physiotherapy on balance and fall rates. The pooled estimate of the impact of physiotherapy in this review demonstrated a significant enhancement in balance. However, the authors did not discover adequate proof to help or discredit the impacts of physiotherapy intervention on diminishing fall rate. In another review, Shen et al. [11] examined the effects of exercise training on balance, gait ability, and falls against no intervention and placebo intervention. The authors concluded that there was no evidence that training decreased the number of fallers over the short or long term (*p* > 0.05).

Regardless of the deficient proof given by aforementioned research, proof has risen out of a different review that has concentrated on the potential advantages of physiotherapy for individuals with PD, and a few advantages regarding balance and other fall-related variables have been accounted for. For instance, Kwakkel et al. and Goodwin et al. [12, 13] found that the utilization of physiotherapy gave promising outcomes, being valuable regarding balance**,** gait velocity, and muscle strength. In any case, the poor methodological nature of the studies incorporated into these reviews demonstrations to restrain both these reviews and the developed proof. In this manner, leading a review that centers around fall and incorporates just randomized controlled studies to decide if physiotherapy is gainful for fall chance decrease in individuals with PD is considered poor.

A systematic review is increasingly confined in center and tries to answer specific research inquiries from the available literature. However, the scoping review is a procedure intended to deliberately recognize the expansiveness of writing in a region being researched [1]. The intention of this paper was to scope the physiotherapy practice/role to prevent fall in patients with PD.

## Methods

A scoping review was conducted including determining the research question; determining relevant studies and study selection; charting the data; and finally collating, summarizing, and reporting the findings. The main phase of searching included choosing papers based on the title and abstract. When every one of the titles was chosen, duplicates were removed. Irrelevant studies were disregarded. If there was any vulnerability about the significance of a study, the entire paper was read. The search strategy was modified to suit different databases. The following electronic databases were searched in May 2019: Medline, Amed, Cinahl, PubMed, PsychInfo, Cochrane Library, and Physiotherapy Evidence database (PEDro), and the search was expanded with the following references lists from selected articles.

The design of the study (controlled randomized trial), participants (people with PD), type of the treatment (compare the effect of a physiotherapy intervention to any comparable including other physiotherapy intervention), type of outcomes (report the effect of physiotherapy on fall and/or at least one fall risk factor), language (written in English), and availability of the full text were important and needed to be considered to find the most relevant studies.

The researcher independently extracted data from the studies by using a standard data extraction form. An outline of all the included material was condensed in a table that maps the literature. The literature was organized and presented in relation to the author, location, and year of publication; aim; method; outcome measures; and results.

## Findings

**Fig. 1:**
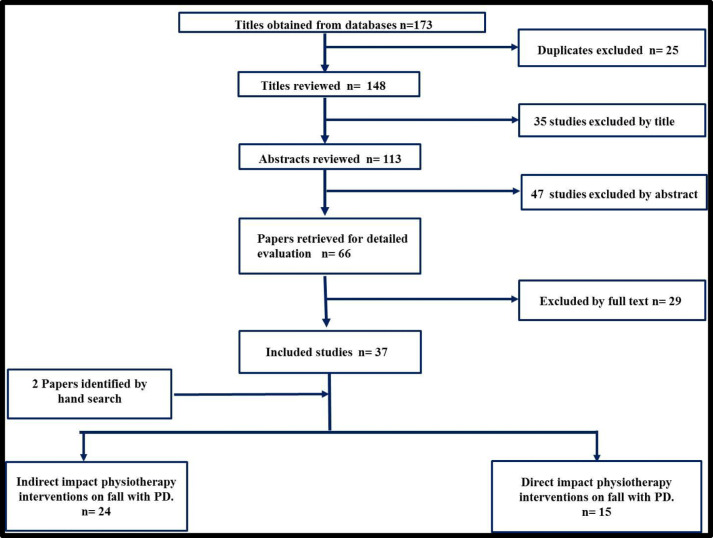
Flowchart showing the results of the scoping review

A total of 173 papers were identified before 25 duplicates were discarded. Following the screening of the titles and abstracts, 66 studies remained. In total, a further 29 papers were excluded after screening of full text and inclusion criteria, leaving 37 papers eligible for inclusion. Two further papers were identified by hand searching; therefore, in total, 39 papers were included in this scoping review (Fig. 1). Fifteen of the included papers reported the direct effects of physiotherapy interventions on fall and furthermore inspected the effects on other risk factors, whereas the rest of the studies did not examine the benefits of physiotherapy interventions on fall directly. Rather, they announced different variables related with fall, for example, balance, functional mobility, and gait as end points.

### Studies reporting both fall incidence and fall risk factors

The scoping search yielded 15 considered studies containing data important to the direct effect of physiotherapy intervention on fall rate and near fall incidence (Table 1). Different physiotherapy interventions, duration and outcomes were used in these studies.

**Table 1:**
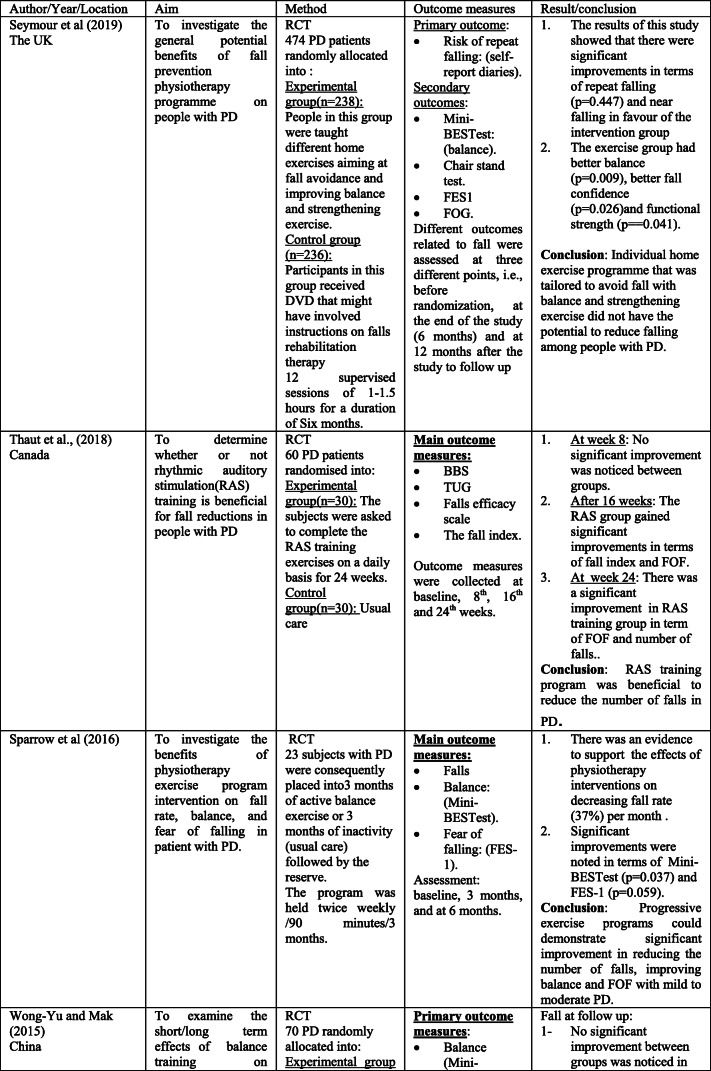

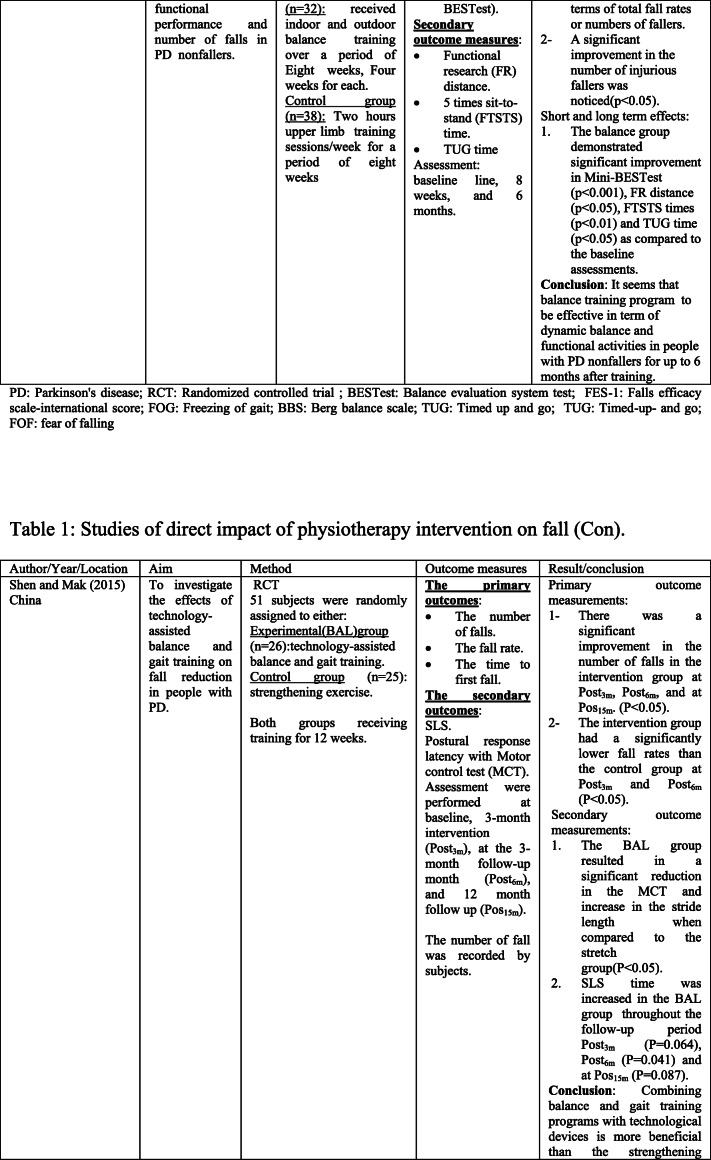

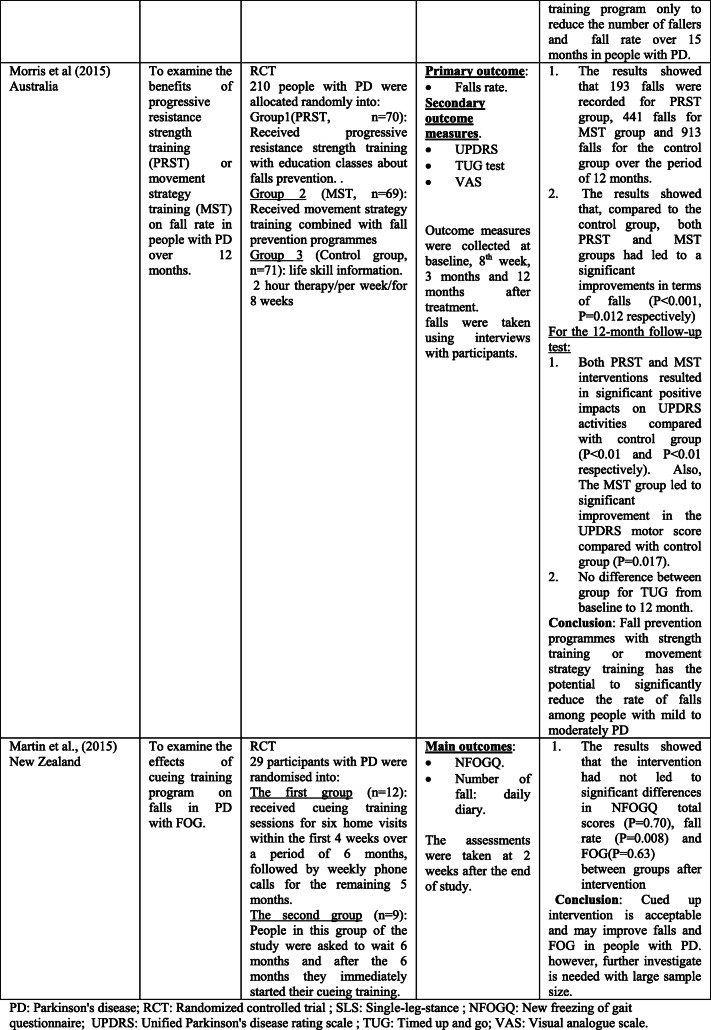

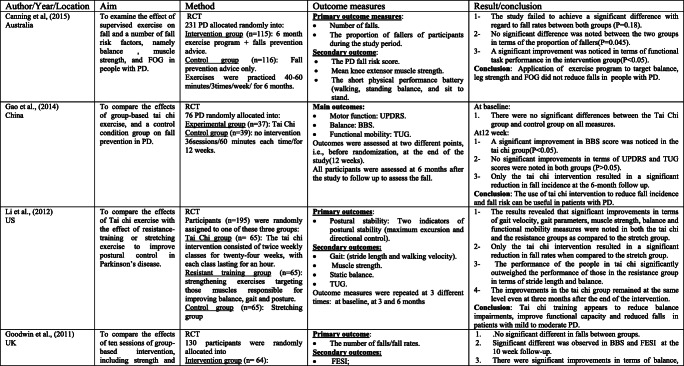

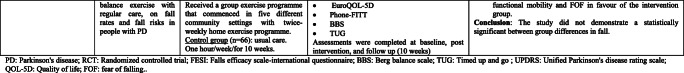

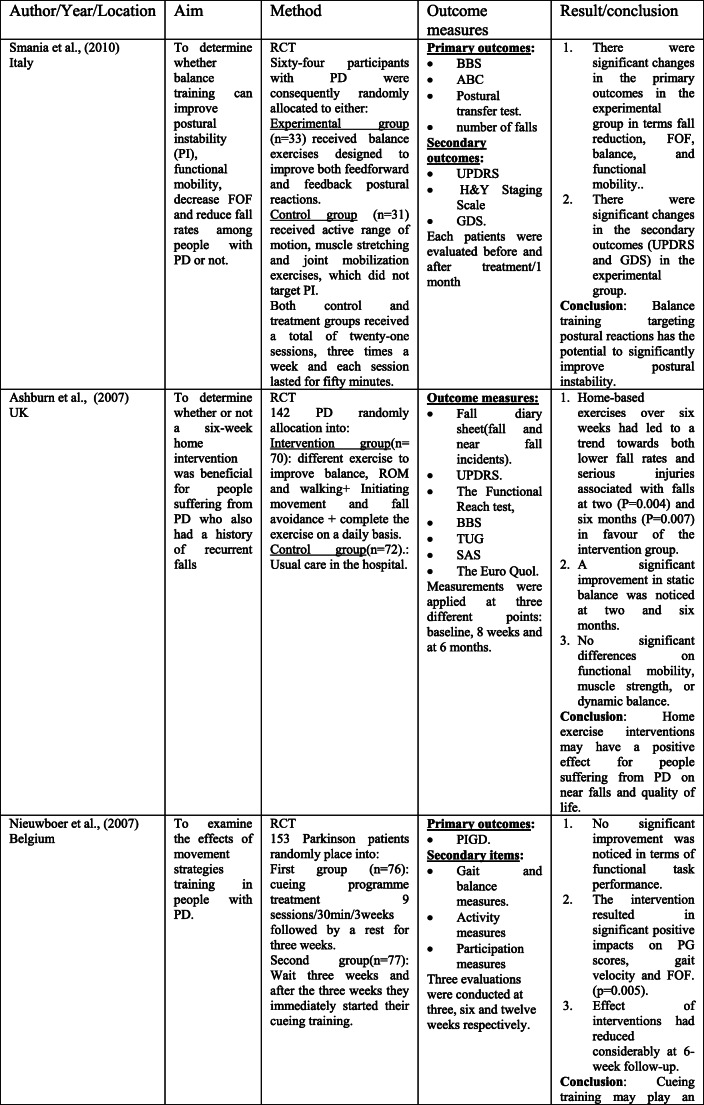

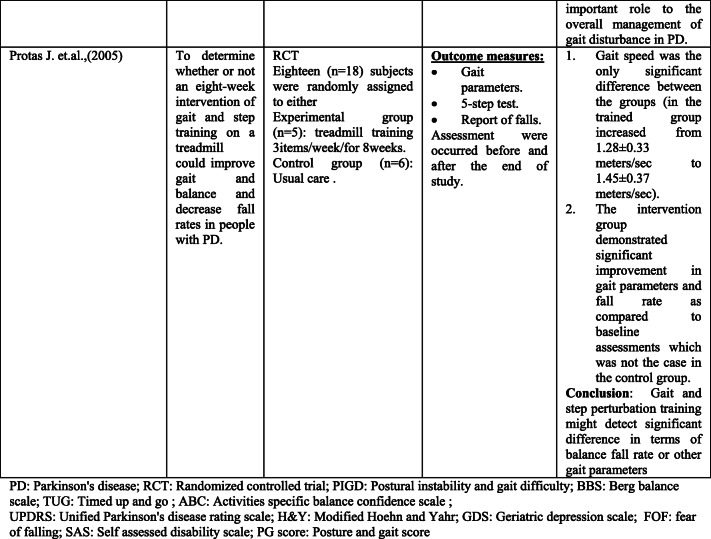
Studies of direct impact of physiotherapy intervention on fall

The sample sizes reported, with included studies, ranged from 18 [14] to 474 [4]. Eight studies [14–18] included 100 participants or less, and only 4 studies [19–22] had an initial sample size of between 130 and 195 participants. Therefore, the rest 3 studies [4, 23, 24] had an initial sample size of between 210 and 474 participants. Every included study in this part recorded the quantity of falls/near fall incidence or fear of falling. These studies recorded falls utilizing falls diary sheet, which is the best gold standard in view of participants/family [4, 15–17, 20–22, 25, 26], telephone interviews [14, 19, 24–26], or medical record observation [23].

Falls were reported at set of intervals, ranging 1-month [26], 8-week [14], 10-week [19]; 3-month [22], 6-month [15, 17, 20, 21, 23, 25], or over 12-month period intervals [4, 6, 24].

These papers also include a variety of interventions related to the field of physiotherapy including traditional physiotherapy exercises, movement, and strategy training. In this way, this paper may give a far-reaching outline about the advantages of physiotherapy as far as fall risk decreases in individuals with PD. For example, the effect of exercise training [4, 14, 19, 21, 24] and balance training exercise [16, 23, 24] for fall prevention in PD has been tested in some studies.

The results of included studies reported that there are no benefits of exercise training for fall preventions [4, 14, 19, 21, 23]. However, some studies reported significant reduction in falls when exercise containing strength and/or balance exercises [16, 18, 24, 26]. Additionally, two randomized trials [20, 27] analyzed the impacts of tai chi exercise on fall decrease in individuals with PD. The discoveries demonstrated that tai chi exercise had positive effects on reducing future falls.

The effectiveness of rhythmic auditory stimulation (RAS) has been resulted in decreasing the number of falls in PD disease [15, 22, 25]. Two RCTs reported that RAS concluded that RAS training significantly reduced the number of falls in PD [15, 22], whereas one RCT study [25] reported that RAS did not have important changes in falls in people with PD.

Furthermore, the interventions of the above studies led to significant improvement in terms of balance [4, 15–17, 19–22, 27] functional mobility [4, 15, 17–20, 23], Fear of falling (FOF) [16, 19, 23, 26], fall rates [14, 17, 18, 21, 24, 25], serious injuries associated with falls [17, 21], freezing of gait (FOG) [4, 22, 25], and gait parameters [14, 15, 17, 18, 20, 22]. Nonetheless, the resulting measures in most of these studies were assessed following the treatment.

Just a couple of studies had follow-up evaluations. However, these studies had short follow-up periods. In this way, it is difficult to give an end with respect to the long-haul impacts of physiotherapy on fall risk reduction. Only three studies had a 1-year follow-up period, and the results showed that strength movement strategy training and multi-dimensional balance training could reduce the rate of falls in people with mild to moderate PD [4, 18, 24].

### Interventions that reported fall risk factors only

Rather than the studies evaluated in the segment above, there are various studies that neither target fallers nor report fall number or close fall rate as results (Table 2). However, these studies examined the impacts of physiotherapy interventions on several factors that are closely associated with fall in people with PD, such as balance, functional task, gait, and muscle strength.

**Table 2:**
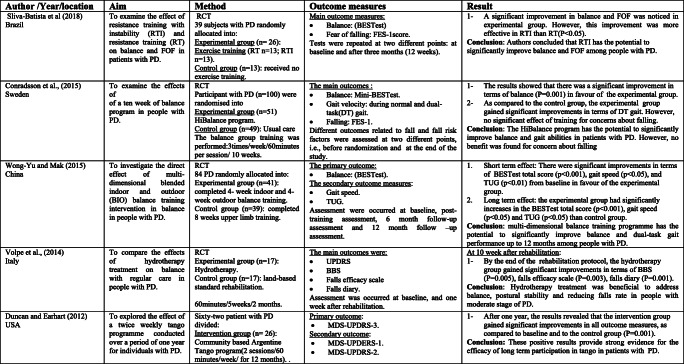

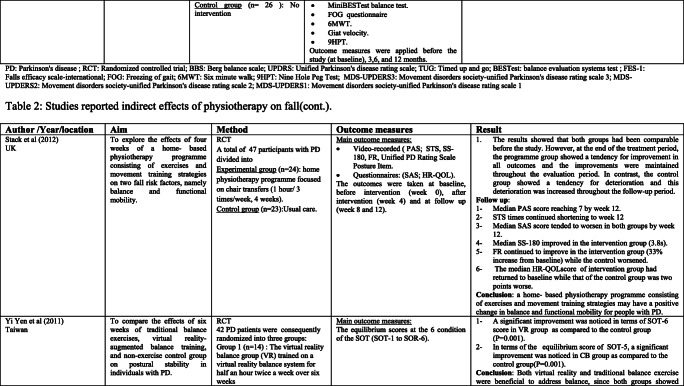

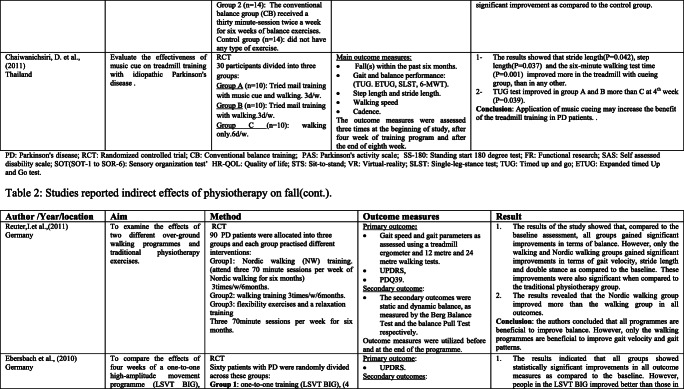

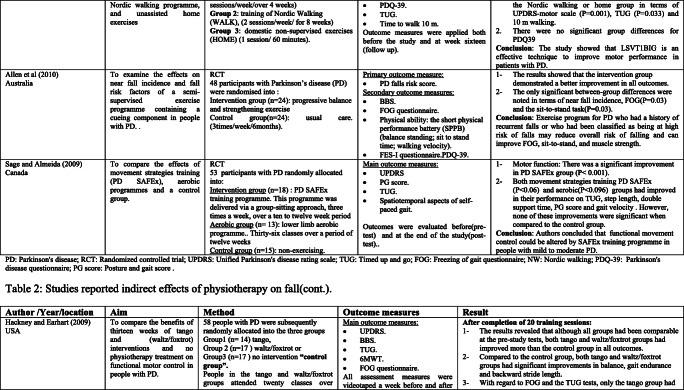

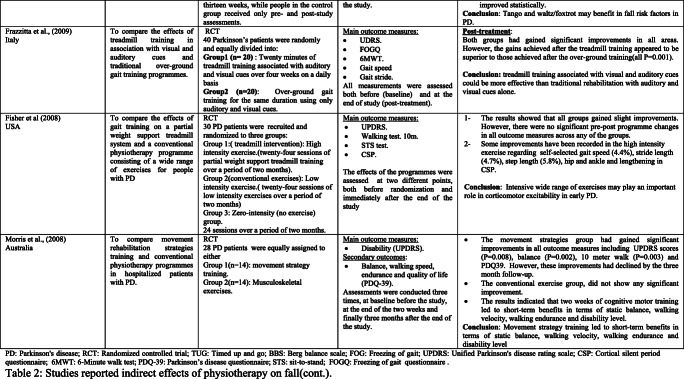

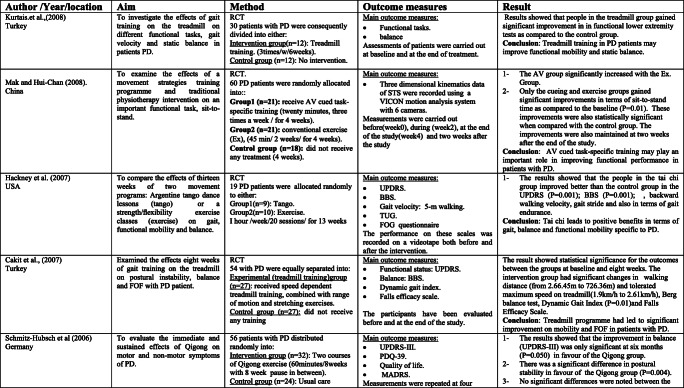

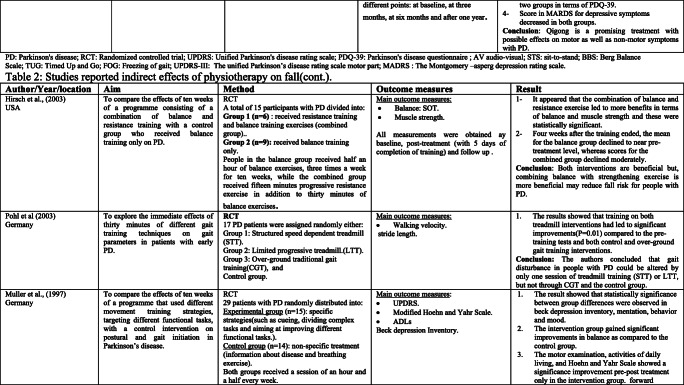

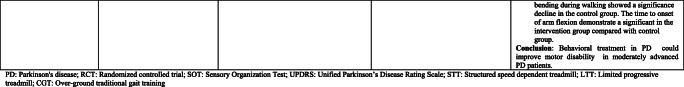
Studies of indirect effects of physiotherapy on fall

### Effect of balance

The significance of balance in fall avoidance is featured. In this scoping review, a few studies detailed the impact of physiotherapy intervention on balance [28–44].

Fourteen studies out of the 17 papers that reported on balance showed physiotherapy intervention had led to a statistically significant improvement in at least one of the balance measures as compared to either control or active interventions [28–34, 36, 37, 39, 40, 42–44]. However, only three studies did not report significant effects [35, 38, 41]. One of the three studies that did not report a remarkable difference between-group used exercise in the comparison intervention [38]. However, this study reported a significant difference as compared to the baseline data within the same group. The second study [41] compared the effects of physiotherapy intervention to a control and found a trend towards improvement in the intervention group, while the control group showed a tendency for deterioration. The third study, by Hackney et al. [35], compared the effects of tango dance or strength/flexibility exercise. The study also reported that balance may be a beneficial to address balance and gait problems specific to PD.

### Effects of functional mobility/performance on functional task

Various studies have analyzed the impact of physiotherapy on functional performance. In this paper, seven studies were reliable in giving the outcomes that physiotherapy interventions lead to a significant improvement in performance on functional task measures as timed up and go (TUG) test, sit-to-stand test, and climbing stairs [34, 37, 38, 41, 45–47]. In light of the after effects of these trials, it gives the idea that physiotherapy is advantageous as far as enhancing functional mobility. However, different physiotherapy programs and different durations were used in these studies. This makes it hard to propose the best kind of physiotherapy to address this issue.

A few authors have contended that, notwithstanding, the motor symptoms associated with PD sufferers are still typically ready to perform complex movements in specific circumstances [36].

Therefore, they suggest using movement strategies training to compensate for and bypass the deficits in the basal ganglia. Three studies used movement strategies training either as a stand-alone intervention or combined with other exercise, and each showed positive results [38, 44, 46]. However, other studies examined the effects of programs that do not include movement strategies and showed positive results [34, 37]. These programs include tango or treadmill. Thus, it makes it difficult to pinpoint which type of physiotherapy is more effective.

### Effect on gait/muscle strength

Fifteen studies included in this part of scoping review reported outcomes related to gait deficits and showed promising results [29, 34–40, 42, 46–51]. The most result measure used to assess gait quality in these studies is gait velocity over a short distance. Ten of which indicated significant enhancement on account of the physiotherapy [29, 34, 36–38, 40, 42, 47, 48, 51]. Other gait results, for example, stride length and walking endurance, were additionally assessed in a few studies and were found to enhance with physiotherapy [34, 36, 48, 49].

Findings emerging from the results of the reviewed studies, related to gait and fall risk, are that people suffering from fear of gait (FOG) are likely to benefit from physiotherapy. However, this finding rises out of a predetermined number of studies.

Just six trials provided details regarding gait freezing [34–36, 40, 44, 48]. Three out of the six trials demonstrated positive results for physiotherapy when contrasted either with control or with some other active intervention [40, 44, 48]. The lack of significant improvements in the other three studies does not necessarily indicate the absence of the benefits of physiotherapy. To illustrate, all of these studies did not target FOG specifically and did not include such a problem as an inclusion or exclusion criterion.

### Effect of muscle strength

It appears that muscle strength may likewise enhance after physiotherapy in individuals with PD [44]. Hirsch et al. [31] compared the benefits of two physiotherapy interventions on two fall risk factors, namely balance and muscle strength. In this study, Hirsch et al. [31] compared the effects of 10 weeks of a program consisting of a combination of balance and resistance training with a control group who received balance training only. The results of this study were promising and showed that both groups gained significant improvements in all outcomes, and these gains were maintained for at least 4 weeks. However, the program of this study mainly focused on muscle strength, whereas strengthening training, in another study, represents a small portion of the programs. For example, Allen et al. [44] used resistance training as a component of a program that included cueing strategies and balance training. Therefore, it might be argued that the dose of the strengthening exercise was not sufficient enough to produce change in muscle power.

## Discussion

### Direct impact of physiotherapy interventions on fall

Even though falling is predominant among those experiencing PD, little work has been done to prevent this issue. Only 15 studies examined the direct effect of physiotherapy in fall in this specific population. The sample size was small in most of the inspected studies. Besides, the subsequent periods in the included studies were moderately short, with most of the studies looking at the impacts of physiotherapy immediately after the end of the interventions.

However, this paper represents the accessible studies that inspect the advantages of physiotherapy for individuals with PD. The immediate effects of physiotherapy in fall were estimated in these studies in terms of fall number and near fall incidence. These results were gathered fundamentally utilizing a self-reported fall diary sheet that was finished by participants themselves or medical staff. However, although fall diaries are considered valid and a reliable way to record fall incidence, factors such as participants’ ability to recall fall incidence, education level, and native language may affect the accuracy of the recording of fall in such diaries, and this should be considered when using this approach [52]. Despite this, the consequences of this scoping review propose that physiotherapy intervention may be useful in decreasing the quantity of falls and near fall incidence in individuals experiencing PD [15–17, 20, 21, 23, 25, 26]. This suggestion is based on limited evidence. Eleven studies showed that physiotherapy resulted in a reduction of fall rate [14, 15, 17–21, 23, 24, 26]. Yet, this reduction was significant only in seven studies [4, 15, 18, 20, 23, 24, 26]. Also, one study reported near fall incidence as outcomes and showed significant reduction [21]. The absence of the significant decrease in the number of falls in this study does not really demonstrate the absence of the impacts of physiotherapy. Rather, it may indicate the lack of adequate sample size to detect this change statistically, as noticed in the studies conducted by Protas et al. [14] and Martin et al. [25]. Another conceivable explanation behind the absence of significant improvement in terms of fall rates is the absence of incorporating appropriate intensity and type of training. Observably, every one of the interventions that did not reveal remarkable improvement involved interventions of moderately brief length or exercises that did not challenge balance, while the proof rising up out of studies concerning the older population in general recommend a higher dose of exercise and activities that challenge balance to achieve a positive impact on fall rate [5, 44]. Sherrington et al. [5] conducted a systematic review and meta-analysis and indicated that approximately half of the fall incidence among old people can be prevented by a well-designed program with a dose of at least 50 h over the trial period. Additionally, they brought up that intervention, including exercise, that challenges balance capacities are bound to counteract fall more often than those intervention comprising of different sorts of activities, for example, walking or strengthening exercises.

Therefore, it is not surprising when a significant reduction in fall number was noticed in the studies [20, 26, 27] that incorporated a tai chi exercise program with a high dose (1 h, twice weekly for 6 months, > 50 h). The tai chi exercise is also considered a balance-based exercise.

Similarly, Smania et al. [26] used a variety of exercises that greatly challenge balance in different situations including standing, walking, and when doing functional activities, and a significant reduction in fall numbers was demonstrated. However, the dose of this program was 1 h twice weekly for 7 weeks, which was lower than the recommended dose (< 50 h). Thus, it might be argued that interventions consisting of only balance exercises that exceptionally challenge postural control in various circumstances may be a decent system to prevent fall in individuals with PD, even with the portion lower than the suggested dimension. However, this needs further examination. Another related finding is that programs, for the most part contain and fortify preparation, appear to be not viable in terms of fall rate decreasing in individuals with PD. Some included studies exploring the advantages of a strengthening exercise program and demonstrated that the strengthening activity had not caused any significant decrease in fall occurrence in correlation with the control group [20, 24, 25].

It is consistent with several systematic reviews concerning fall in general in older people. For example, Sherrington et al. [5] conducted a systematic review of 44 controlled randomized trials and showed that strengthening exercise alone is not beneficial for old people who suffer from recurrent falls. Other review has been conducted by Shen et al. [11] who showed that no clear evidence that training exercise decreased the number of fallers in PD over the short- or long term.

In addition, due to the limited number of trials reviewed in this paper which report the direct impact of physiotherapy on fall, these findings should be interpreted with caution. Furthermore, more control randomized trials with large sample sizes are required both to affirm these discoveries as well as to locate the intensity and type of exercise. Additionally, it should be clarified that all these trials included individuals with PD who did not have other neurological conditions and who were mentally and medically stable. In this way, generalization of these findings to other populations is not advisable.

### Indirect effects of physiotherapy on fall effect on composite score

Recruiting many people can prove difficult to achieve, especially when researchers target a specific population such as people with PD [44]. For this situation, composite score is a decent technique to be used [44]. Composite score can be utilized to demonstrate the risk of falling, and the consequence of the reviewed trials shows that physiotherapy may positively affect such a measure. In this paper, two studies used a composite score (PIGD) that includes items related to gait and balance [22, 46]. These two studies exhibited that physiotherapy interventions lead to significant positive effects on such a score, when contrasted with usual care. Nonetheless, there is no one study that examined the sensitivity of this measure to foresee future fall. Then again, one study focused on fallers utilizing a composite score, PD fall risk score, that incorporates commitment that includes weight from muscle strength, balance, and FOG [44]. The authors in this study cited the validity and reliability of this measure. However, they found that physiotherapy did not result in any significant improvement in this score when compared to usual care.

### Effect on balance

Considering the consequences of the studies included in this paper, it appears that different types of physiotherapy can possibly enhance balance. These types include traditional balance training, virtual reality balance training, resistance exercise, tai chi, movement training strategies, treadmill training, and multi-dimensional interventions. In any case, because of the various number of measures utilized, and the limited number of studies that analyze the impacts of various physiotherapy intervention on balance, it is not clear what sort of physiotherapy is the most beneficial for balance. A systematic review conducted by Addison et al. [53] contend that the type of physical activity may lead to an improvement in balance performance. Although this discovery supports the use of physiotherapy, the degree of the advantages is not known. Accordingly, future studies should address this impediment and focus on identifying the best intervention to target balance impairment in people with PD.

### Effects on functional mobility/performance on functional tasks

Difficulty in performing functional tasks is one of the problems associated with PD and is strongly associated with fall. Measures intended to assess functional performance can be utilized to assess either balance or fall risk in individuals with PD. Consequently, various studies [4, 19, 20, 26, 29, 34, 37, 45] have analyzed the impact of physiotherapy on such results. In view of the results of these trials, it gives the idea that physiotherapy is advantageous regarding enhancing functional mobility. However, distinctive physiotherapy programs and diverse duration were utilized in these studies. This makes it hard to propose the best kind of physiotherapy to address this issue.

### Effects on gait

Abnormal gait is, with no uncertainty, one of the elements that is unequivocally connected with fall and balance in individuals with PD. In this paper, distinctive interventions related to gait deficits were utilized in the included studies and showed promising results. These interventions were various, going from movement strategies training to daily walking at home. Therefore, extracting a message about the best method to improve gait speed, stride, and endurance is difficult. Be that as it may, it creates the impression that interventions involving walking exercise or movement strategy training are more valuable than those without [36, 51]. Additionally, the results of the reviewed studies suggest that treadmill training, nordic walking, and amplitude movement training are better than traditional over-ground gait training [38, 46, 48]. However, these recommendations depend on a set number of studies and need further investigation.

### Effects on muscle strength

Lower limb muscle strength is an important fall risk factor. It has been found that there is a strong relationship between lower extremities’ muscle strength and fall risk in people with PD [15]. This is normal because these muscles play a role in maintaining static and balance in people [44]. Moreover, there is a connection between muscle strength and gait patterns and walking ability which are also considered fall risk factors in people with PD [50]. In view of the consequences of the studies incorporated into this paper, it appears that muscle strength may likewise enhance after physiotherapy in individuals with PD [4, 16, 20, 28, 31]. In any case, it ought to be viewed that the aftereffects of the included studies were conflicting and future examinations may change this case. To illustrate, four studies reported on muscle strength, two of which found significant improvement as compared to either no physiotherapy treatment or an active intervention [20, 31], while two studies did not find significant gains [21, 44]. These studies had programs containing resistance training. Nonetheless, intensity was different among the trials. The studies that showed critical enhancement had programs mainly focusing on muscle strength, while strengthening training, in alternate studies, represents a small portion of the programs. Consequently, the effect of physiotherapy on muscle strength should be further investigated.

### Effects of physiotherapy on FOF

Fear of falling, or FOF, is additionally one of the fundamental contributing elements to the beginning of fall in individuals with PD, and the discoveries of this paper support the use of physiotherapy to target the fear of falling. Four studies analyzed the effect of physiotherapy intervention on this fear, and these studies demonstrated critical positive outcomes for physiotherapy [19, 22, 26, 33]. Distinctive interventions were utilized in these studies, including cueing training, balance exercise, and treadmill training. Detectably, every one of the trials revealed enhancement in fear of falling and furthermore indicated enhancement in balance. This may show that impaired balance is a fundamental driver for the beginning of the fear of falling, and along these lines, any enhancement as far as balance would prompt a lessening in this fear. In this way, it tends to be contended that any intervention is gainful because of a paranoid fear of falling if it enhances balance. This finding is like the evidence emerging from systematic reviews that concern the effects of physiotherapy on elders in general. For instance, Zijlstra et al. [54] reviewed 11 controlled randomized trials and indicated that different kinds of exercise programs, such as tai chi exercise and balance training, have the potential to improve the fear of falling in elderly people living in the community, although these studies were not planned explicitly to treat the fear of falling.

## Conclusion

Falling is one of the complications related with PD that effects up to 83% of sufferers. It creates the impression that this issue is likewise repetitive. Subsequently, a generous number of studies have been led to distinguish the factors that contribute to the onset of fall in this population and consequently several factors have been illustrated. These factors include previous fall history, impaired balance, gait problems, muscle weakness, and poor performance in functional activities, depression, and FOF. However, little work has been done in terms of fall prevention. The result of this study is promising. In any case, it appears that few controlled randomized trials have not been done to examine the direct impacts of physiotherapy on fall. Despite this, there is proof that physiotherapy intervention may be useful in diminishing fall rate and near fall incidence among individuals with PD. However, the components of the interventions play a main role in the outcomes. According to the results of these studies, interventions that highly challenge balance are likely to have positive impacts on fall. However, more research is needed to support or refute this finding. Furthermore, the results were also promising in that physiotherapy interventions have a positive impact on fall risk factors. Factors such as balance, functional mobility, FOF, and muscle strength may improve by physiotherapy. However, precisely the best interventions to focus on these factors stay questionable as do the long-term impacts of physiotherapy. One of the limitations in this paper is that most of the included studies barred individuals with other mental and neurological infections. In this manner, the generalizability of the discoveries cannot be accomplished for those with PD and other cognitive impairments or different diseases. Furthermore, this scoping review aims of displaying trends in the literature without giving distinction to methodological quality or empirical “weight” [55].
